# Quercetin-Rich Extracts from Onions (*Allium cepa*) Play Potent Cytotoxicity on Adrenocortical Carcinoma Cell Lines, and Quercetin Induces Important Anticancer Properties

**DOI:** 10.3390/ph15060754

**Published:** 2022-06-16

**Authors:** Alan A. Veiga, Ana Carolina Irioda, Bassam F. Mogharbel, Sandro J. R. Bonatto, Lauro M. Souza

**Affiliations:** 1 Instituto de Pesquisa Pelé Pequeno Príncipe, Curitiba 80250-060, Brazil; alanwessels6@gmail.com (A.A.V.); anairioda@gmail.com (A.C.I.); bassamfm@gmail.com (B.F.M.); sandrobonatto@gmail.com (S.J.R.B.); 2 Faculdades Pequeno Príncipe, Curitiba 80230-020, Brazil

**Keywords:** cancer, antitumor agents, flavonoids, H295R, SW-13, onions, phytochemicals

## Abstract

Adrenocortical carcinoma (ACC) is a rare subtype of cancer, with a poor prognosis in children and adults. Mitotane is the only approved adrenolytic drug for the treatment of ACC, which has controversies regarding its efficacy and side effects on patients. Onion (*Allium cepa*), a worldwide consumed food, is associated with many health benefits. Along with its glycosides, the flavonoid quercetin is abundant in onions. After evaluating the cytotoxicity of *A. cepa* extracts on adrenocortical carcinoma cell line (H295R), the rich quercetin fractions had better results. Then, we aimed to compare the quercetin vs. mitotane effectiveness, using adrenocortical carcinoma cell lines H295R and SW-13. Quercetin showed a higher cytotoxicity response on both cancerous cell lines after 10 µM concentration, while mitotane only after 30 µM. Cell cycle dynamics were altered upon quercetin treatments, with G2 phase increase with 30 µM of quercetin on H295R cell line and G1 arrest on SW-13 cell line with 15 µM. Early and late apoptosis, alongside intracellular calcium, were increased on SW-13 treated with 30 µM of quercetin, and ROS rates were reduced by quercetin on H295R. Therefore, quercetin-rich onions have the potential to be a natural source of anticancer agents for adrenocortical carcinoma.

## 1. Introduction

Cancer consists of a group of diseases that present abnormalities with normal cell cycle dynamics, making them unable to control their proliferation. After turning into cancerous cells, the growing tumor becomes capable of recruiting other noncancerous cells to form the so-called “tumor microenvironment”, stimulating the generation of new blood vessels for nutritional demands and able to spread around the organism, generating metastasis. These important characteristics, common in various cancer subtypes, are included in the so-called “hallmarks of cancer” [1,2].

Adrenocortical carcinoma (ACC), including a group of less common cancers, is a malignancy that originated in the cortex region of the adrenal glands, responsible for the synthesis of steroidal hormones. ACC is an extremely aggressive cancer, with low survival rates among those patients at advanced stages, even after one or more surgical interventions [3]. In southern Brazil, the incidence of ACC is unusually higher than in other world regions, particularly affecting children. Investigations discovered a point mutation in the *TP53* gene, called R337H, which contributes directly to the higher incidence of ACC [4].

**Figure 1:**
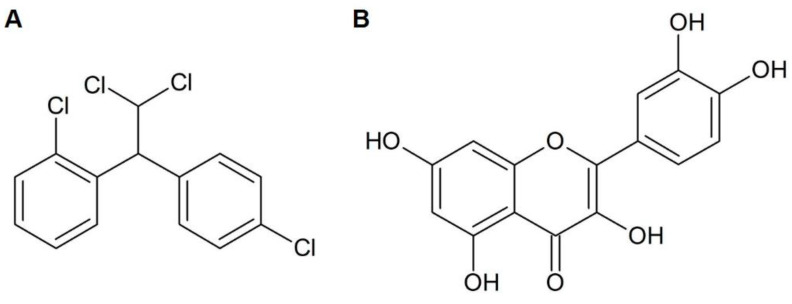
(**A**) Structure of mitotane; (**B**) structure of quercetin.

The only approved drug with considerable specificity to treat adrenocortical carcinoma is the mitotane (o,p′-dichlorodiphenyldichloroethane; Figure 1A), used alone or in combination with other antineoplastic agents. Mitotane is derived from the pesticide dichlorodiphenyltrichloroethane (DDT), and its adrenolytic effect has been reported since 1959 [5]. Nevertheless, difficulties in mitotane administration, such as the insolubility in water and maintenance of the therapeutic range in the bloodstream, remain challenges to be overcome in ACC treatment [6].

To overcome those obstacles, research on new therapeutic alternatives is indispensable. Therefore, the employment of phytochemicals as complementary or first-line treatments for cancer came as a recurring alternative over the years [7,8]. Actually, different plant-derived medicines are currently employed in chemotherapy medication for different cancer treatments, such as the alkaloids vincristine and vinblastine, originally obtained from *Catharanthus roseus*, being used in the treatment of acute lymphocytic leukemia, acute myeloid leukemia, Hodgkin’s disease, neuroblastoma, and small cell lung cancer, bladder cancer, brain cancer, and testicular cancer, among others [9]. Paclitaxel, obtained from *Taxus brevifolia*, is used to treat breast, ovarian, lung, bladder, prostate, as well as other types of solid tumor cancers [10]. Nevertheless, plant-based medicines also include many other different drugs employed for different diseases other than cancer.

*Allium cepa* L., commonly known as onion, is a commonly consumed food worldwide, with antioxidant effects related to its intake. Among its biological active molecules, flavonoids are appointed as the major contributors to this beneficial effect [11]. Flavonoids are a type of plant secondary metabolite largely known for their antioxidant effects [12]. An important member of the flavonoid family is quercetin (3,3′,4′,5,7-pentahydroxyflavone; Figure 1B), one of the major phenolic compounds consumed in the vegetable-based diet [13]. Quercetin has been appointed as an anticancer compound, especially for apoptosis induction, metastatic inhibition, and antiproliferative actions. Its effects were observed on a wide array of cancer subtypes, including breast, lung and prostate [14,15,16]. Along with quercetin, onion has other flavonoid derivatives, notably the glycosides from quercetin which can also exert beneficial health properties [17,18].

Thus, due to the lack of investigations attempting to discover novel antineoplastic agents for treating adrenocortical carcinoma, and the potential of *A. cepa* as a source for different bioactive compounds, especially in oncology research, we performed different in vitro assays in order to evaluate the effectiveness of the extracts and fractions from different onion layers, using the well-established steroidogenic H295R and the non-steroidogenic SW-13 as adrenocortical carcinoma cell models. In comparison to mitotane, the onion components exert greater cytotoxic effects on these cells, especially those fractions rich in quercetin content.

## 2. Results and Discussion

### 2.1. Composition Analysis of Allium cepa Extracts and Fractions

Onions are ubiquitously cultivated around the world, and they are present in the daily diet of many people, the consumption of which varies from eating its leaves to using the bulbs as the basis for sauces [19]. As a nutrient source, it can provide a wide array of bioactive compounds, varying from saponins and peptides to a large amount of carbohydrates [20]. Its usage in antioxidant and anticancer research relies mostly on its phenolic compounds content.

**Figure 2:**
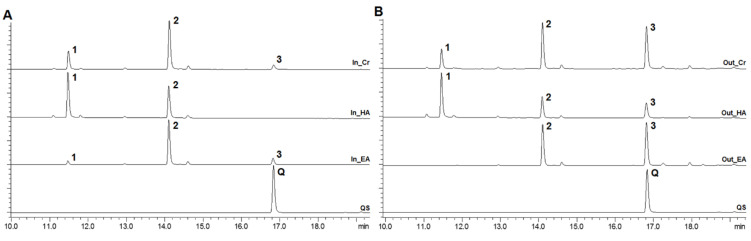
Chromatograms of *Allium cepa* inner (In) and outer (Out) extracts and their respective fractions. (**A**) Inner crude extract (In_Cr); Inner hydroalcoholic fraction; (In_HA); Inner ethyl acetate fraction (In_EA) and Quercetin standard (QS). (**B**) Outer crude extract (Out_Cr); Outer hydroalcoholic fraction (Out_HA); Outer ethyl acetate fraction (Out_EA) and Quercetin standard (QS). Peaks 1 and 2 correspond to quercetin glycosides; peak 3 corresponds to quercetin; peak Q corresponds to quercetin standard, at the wavelength of 370 nm.

Different phenolic compounds were described from *A. cepa*, mainly the flavonoids, especially those having the flavonol aglycones and their glycosides, with detaching for quercetin and quercetin-glycosides [21,22,23]. Herein, the onion bulbs were divided into two parts, with the two external layers (outer layer) being separated from the internal parts, named inner layers. This procedure was performed since the literature showed that the phytochemical composition could be different between these layers [24]. Indeed, by the chromatographic analysis, compared with an authentic standard, different concentrations of free-quercetin (peak 3) from both layers were observed, with a predominant amount observed from the extract of the outer layers (Figure 2A,B). Along with free-quercetin, the chromatograms exhibited two other main peaks, and, despite the lack of standards, the other major peaks detected on HPLC analysis are presumably quercetin glycosides, tentatively identified as quercetin-diglucoside (peak 1) and quercetin-glucoside (peak 2) on the basis of the typical UV spectra of peaks 1 and 2, which were quite similar to that of free-quercetin (Figure S1) and similarities to other reports [21,22,23,24,25,26].

The ethanolic extracts were partitioned between water (containing residual ethanol amounts) and ethyl acetate. Whereas the hydroalcoholic fraction was concentrated, mainly, the diglycoside, the free-quercetin and its monoglycoside were concentrated in the ethyl acetate fraction from both extracts, with detaching to the outer layer extract, with the highest concentration of free-quercetin (Figure 2B), which was quantified through a calibration curve (R^2^ 0.999) using an authentic standard of quercetin (Table 1).

### 2.2. Cytotoxicity on Adrenocortical Carcinoma Cell Line

**Figure 3:**
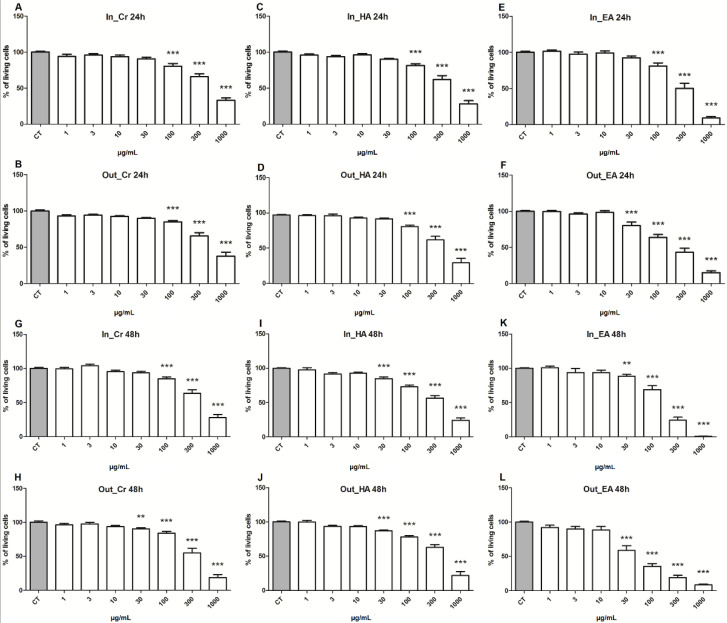
Cytotoxicity of *Allium cepa* extracts and its fractions after 24 and 48 h of treatment on H295R cell line. *(***A**,**G**) Inner crude extract (In_Cr); (**C**,**I**) inner hydroalcoholic fraction (In_HA); (**E**,**K**) inner ethyl acetate fraction (In_EA); (**B**,**H**) outer crude fraction (Out_Cr); (**D**,**J**) outer hydroalcoholic fraction (Out_HA); (**F**,**L**) outer ethyl acetate fraction (Out_EA). CT Control. ** (*p* < 0.005); *** (*p* < 0.0005) in comparison with respective controls.

The cytotoxicity was evaluated with the MTT assay of all extracts and fractions on the adrenocortical carcinoma cell line, H295R, upon 24 and 48 h of treatment (Figure 3). The results confirmed that both layers (inner and outer) have toxicity to this adrenocortical carcinoma cell line, both being statistical significant in concentrations from 100 µg/mL in 24 h (Figure 3A,B), with a slight difference after 48 h, where the crude extract from the outer layer (Out_CR) was better, with a cytotoxic response at concentrations from 30 µg/mL (Figure 3G,H). Considering that the difference between the extracts was, mainly, the amount of free-quercetin, with higher concertation in the outer layer, both extracts were fractionated by liquid phase partition, making it possible to concentrate the free-quercetin in the ethyl acetate fractions, although the highest concentration was from the outer layer (Out_EA).

For all investigated fractions, a significant cytotoxic effect on tumor cells was observed, especially in concentrations higher than 100 µg/mL within 24 h of treatment, and, after 48 h of incubation, cytotoxic effects were observed at concentrations from 30 µg/mL. Nevertheless, the ethyl acetate fraction from the outer layer (Out_EA) exhibited a better cytotoxicity action at a concentration from 30 µg/mL in the first 24 h (Figure 3F). This good result was confirmed after 48 h of treatment, where the cytotoxicity observed showed that the fraction Out_EA had the greater activity on the H295R cells, with the concentration of 30 µg/mL inducing an expressive loss of the cell viability, corresponding to 41.39% ± 6.83 (Figure 3L). Compared with other extracts or fractions, similar results were obtained only with 100 µg/mL of In_EA (31.31% ± 6.03) or 300 µg/mL of Out_Cr (44.96% ± 7.03). These results suggested that quercetin and its two main glycosides may be good candidates for evaluating its anticancer properties.

Ethyl acetate (EA) fractions from both inner and outer layers exhibited a higher cytotoxic potency against H295R tumor cell line, especially the EA fraction obtained from outer layers. Along with a quercetin-monoglycoside, the fractions EA have concentrated the free-quercetin, mainly in the Out_EA, the fraction with the best cytotoxicity evaluated, suggesting that quercetin is an important active component from *Allium cepa*, and therefore, it merits further investigation. Since the commercial standard of quercetin is easily found, we opt to carry out a deeper investigation of the cytotoxic mechanisms using a standard of quercetin (Q4951; ≥95% HPLC; Sigma-Aldrich^®^ St. Louis, MO, USA). Since with the treatment of 48 h, the effects were enhanced, in comparison to 24 h, all following assays were performed with 48 h of treatment.

### 2.3. Quercetin Plays Higher Cytotoxicity Than Mitotane

**Figure 4:**
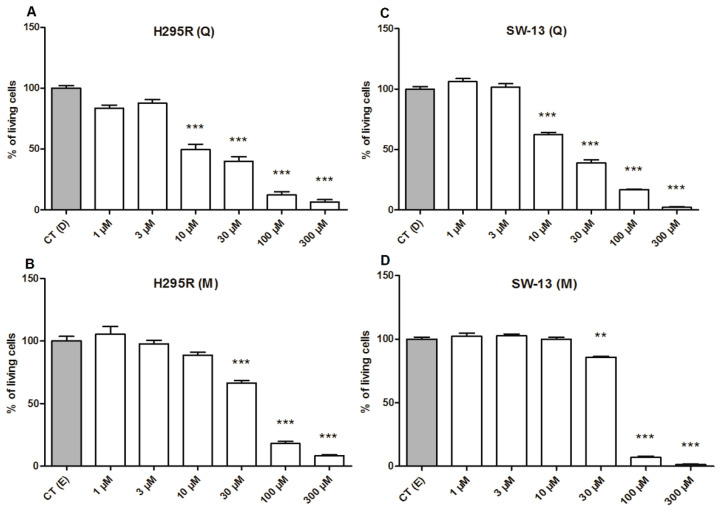
Cytotoxicity of quercetin and mitotane on the adrenocortical carcinoma cell lines. (**A**,**C**) Effect of quercetin (Q) on cellular viability measured by the MTT assay after 48 h of treatment for H295R and SW-13 adrenocortical carcinoma cell lines; (**B**,**D**) effect of mitotane (M) on cellular viability for H295R and SW-13 cell lines. ** (*p* < 0.005); *** (*p* < 0.0005) in comparison with respective controls.

In order to investigate the antitumor potential of the main compound found in the most active fraction of *A. cepa*, the quercetin was assayed against two adrenocortical carcinoma cell lines, the former investigated H295R and, now, the ATCC SW-13 cell line. The initial antitumor assays were obtained by the cytotoxicity, evaluated with the MTT assay, after 48 h treatment, setting the correspondent vehicle controls as 100% of viability. The results confirmed quercetin as an important active compound from the *A. cepa* extracts, since an expressive loss in the viability of both tumor cell lines, H295R (Figure 4A) and SW-13 (Figure 4C), were observed with low concentrations of quercetin, that at 10 µM promoted a decrease in viability on H295R cells of 50.41% ± 2.53 and, on SW-13 cells, of 37.34% ± 3.05. At 30 µM, the loss in viability achieved 60.12% ± 3.72 on H295R cells, similarly to SW-13, which reached 60.97% ± 2.33. By comparison, the drug mitotane was able to decrease cellular viability only with concentrations higher than 30 µM, reaching 33.47% ± 2.01 for H295R cells, and for the less sensitive cell, SW-13, at 30 µM, only a less significant loss in the viability was observed (9.42% ± 1.84). To an epithelial non-tumor cell line, MCF-10A, used as a control, harmful toxicity effects were observed only at concentrations from 100 µM, with a viability loss of 82.73% ± 3.07 for quercetin and 36.46% ± 2.63 for mitotane (Figure S2A,B). Further assays were performed only with these two cancer cell lines.

To ensure the cytotoxicity of quercetin on the adrenal carcinoma cell lines, we performed a second methodology, evaluated by the crystal violet assay. Compared with respective controls, on H295R cell line, quercetin induced a significant decrease in the cell viability, at concentrations from 10 µM, whereas mitotane exhibited toxicity only at 30 µM (Figure S3A,C). The SW-13 cells, treated with quercetin showed loss of viability at concentrations from 30 µM, whereas 100 µM of mitotane was necessary to promote the loss of viability (Figure S3B,D). 

Although other *A. cepa* components can also exert cytotoxicity against tumor cells, the free quercetin, present mainly in the ethyl acetate fractions, seems to be the main responsible for cytotoxic effects observed in extracts and fractions of *A. cepa*. Indeed, by using the commercial standard of free-quercetin, we could confirm that quercetin is a potent cytotoxic agent against ACC cells, with higher effects than mitotane, the standard drug used for treating ACC patients. A common impairment of mitotane therapy relies upon how much the patient needs to intake the drug to achieve plasmatic levels, within the considered therapeutic range, alongside with its variable half-life, ranging from 18 to 160 days [27]. The plasmatic concentration of mitotane, most accepted as therapeutic, ranges between 14 mg/L and 20 mg/L (~40–60 µM). However, different side effects, such as nausea, diarrhea, skin rash, weakness, and confusion, are associated with the use of mitotane, with severe ones such as neurotoxicity often observed with blood concentrations above 20 mg/L [28]. On the other hand, supplementation with quercetin at 1.4 g/m^2^ of body weight in cancer patients, within intervals of 3 weeks, was not related to any severe side effects [29].

Therefore, after cytotoxicity evaluations, further in vitro assays were performed with three defined concentrations of quercetin, at 30 µM (high) 15 µM (intermediate), and 5 µM (low), and mitotane was subsequently used at a single concentration, at 30 µM, equivalent to the higher concentration of quercetin.

### 2.4. Cell Cycle Dynamics and Proliferation Rates Show Different Distributions between Cells and Treatments

**Figure 5:**
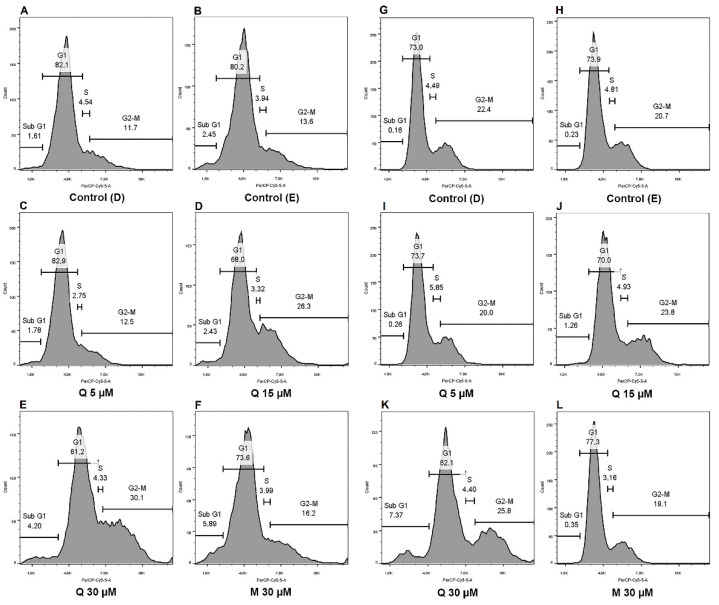
Quercetin and mitotane exerts different effects upon cell cycle after 48 h of treatment. (**A**–**F**) 7-AAD staining for the analysis of cell cycle dynamics on H295R cells; (**G**–**L**) 7-AAD staining for the analysis of cell cycle dynamics on SW-13 cells.

Treatment with 30 µM of quercetin on H295R promoted a decrease in G_1_ (65.40% ± 2.19) with an increase in G_2_/M phases (25.53% ± 2.78) (Figure 5A; Table 2). Treatment with quercetin at 15 µM and 30 µM on SW-13 cells promoted a reduction in the G_1_ phase (69.73% ± 0.26 and 64.93% ± 1.41, respectively), and both concentrations were also able to increase the G_0_/Sub G_1_ (1.78% ± 0.26 and 7.52% ± 0.65, respectively) (Figure 5B; Table 2).

Quercetin affected the cell cycle on the SW-13 line, which was arrested in the G_1_ phase by the treatment, with a prevalence of the cells in the G_0_/Sub-G_1_ phases, especially at a concentration of 30 µM. On a regular cell cycle, an important checkpoint is located at the end of the G_1_ phase, which can send the cells to undergo the quiescent/non-proliferative stage called G_0_ [30]. The raising pattern upon G_2_ phase on the H295R cell line with 30 µM of quercetin may be correlated with a hindrance of another cell cycle checkpoint located at the end of the G_2_ phase on a normal cycle, which can determine whether the cell proceeds to mitosis or goes into cell death by apoptosis [31].

**Figure 6:**
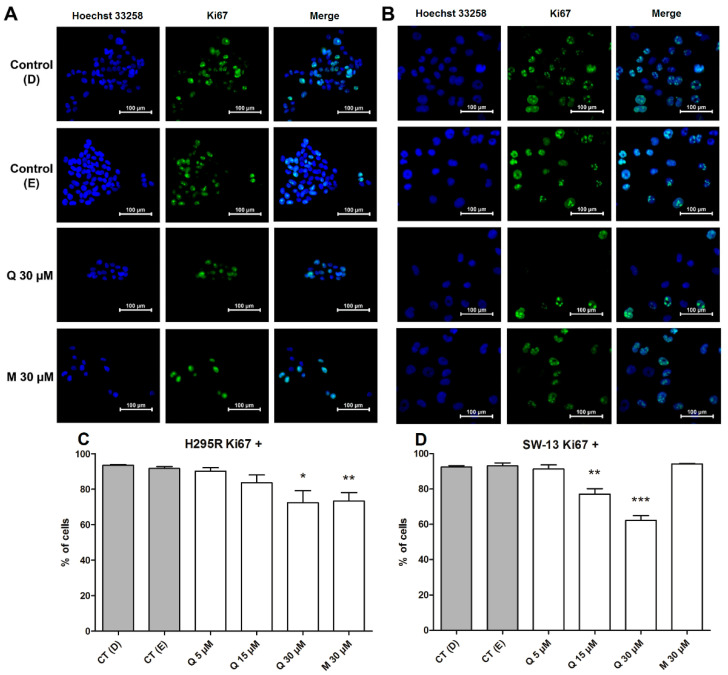
Quercetin and mitotane exerts different effects on proliferation rates after 48 h of treatment. (**A**) Representative images of H295R cells on both controls and treated with 30 µM of quercetin and mitotane; (**B**) representative images of SW-13 cells on both controls and treated with 30 µM of quercetin and mitotane; (**C**) percentage of Ki67-positive cells for H295R cells; (**D**) percentage of Ki67-positive cells for SW-13 cells. * (*p* < 0.05); ** (*p* < 0.005); *** (*p* < 0.0005) in comparison with respective controls.

The cellular proliferation rate was obtained by the Ki67 protein staining assay. The cells were treated with quercetin and mitotane, and those positive for Ki67 were quantified by percentile normalization accordingly, with total cell count by each condition. Although at the higher concentration (30 µM) of quercetin was unable to arrest the H295R cells in the G_0_ phase, it promoted a reduction on the Ki67 positive cell, being statistically different from its respective control (Figure 6A,C). The lowering of Ki67 staining in 30 µM of quercetin on this case may be correlated with the arrest of H295R on G_2_ phase observed on cell cycle analysis (Figure 5A; Table 2). For SW-13 cell line, a reduction in Ki67-positive cells was observed when cells were treated with 15 µM (Figure S4) and 30 µM of quercetin (Figure 6B,D). This result may be correlated with the increase in cells in the G_0_ phase, as observed by the cell cycle assays (Figure 5B), in accordance with a previous study [32]. Quercetin, after 24 h at 100 µM concentration, was able to reduce the expression of Ki67 protein on two distinct nasopharyngeal carcinoma cells [33].

Cell cycle dynamics and proliferation rates are closely related, and both analyses are substantial in the evaluation of cytostatic properties of anticancer substances [34,35]. Quercetin is reported to interfere with the cell cycle dynamics of other cancer subtypes. On breast cancer cell line MDA-MB-453, 100 µM of quercetin on 24 h of treatment was able to promote an arrest in G_1_ phase [36]. In another study, with eleven hepatocellular carcinoma cell lines, quercetin induced multiple effects on the cell cycle, arresting the similar cell strains in different phases, within 48 h of treatment with 25 µM and 100 µM, demonstrating its ability to interfere in this cellular process at distinct ways [37]. Similarly, our results suggest that quercetin at 30 µM played different effects on the cell cycle of both assayed cell lines; nevertheless, the responses observed with quercetin treatment suggested that it could be acting in a cytostatic way on both adrenocortical carcinoma cells.

### 2.5. Quercetin Induced the Early and Late Apoptosis Rates and Increases Free Ca^2+^ Release on SW-13 Cells

The cell death mechanisms were investigated by the fluorescence emitted from H295R and SW-13 cells, stained with both Annexin V and 7-AAD. On the H295R cell line, it was unable to observe significant effects on apoptosis and necrosis rates. Nevertheless, in the SW-13 cell line, treatment with quercetin at 15 µM (2.46% ± 0.22) and 30 µM (15.06% ± 3.75) gave statistically significant results on late apoptosis (Annexin V and 7-AAD positive, staining/Q2, Figure S5J), whereas the early apoptosis rates (Annexin V positive staining/Q3 Figure S5K) were augmented only when cells were treated with quercetin at 30 µM (53.76% ± 18.24). Necrosis, as observed by 7-AAD-positive staining, did not raise at significant levels for both cells and treatments. All data were plotted on quad charts (Figure S5), with the data summarized in Table 3.

According to previous studies, although with different cell lines, quercetin was able to increase apoptosis rates with variable concentrations. On B-16 melanoma cells, quercetin (50 µg/mL) could increase the apoptosis rates after 48 h of treatment, with similar levels to control drug etoposide [38]. In 12 h of treatment with 50 µM and 100 µM of quercetin, the flavonoid augmented the apoptosis rates on HL-60 leukemia cell lines, even with the presence of caspase inhibitors [39]. Despite our cytotoxicity assays demonstrating that 10 µM of quercetin on MTT assay were close to 50% of viability loss and 30 µM reduced the cell count by half on crystal violet assay, our results on apoptosis analysis with the H295R cell line were not conclusive. This might suggest that quercetin could be leading the H295R cell to a distinct death pathway than apoptosis. 

**Figure 7:**
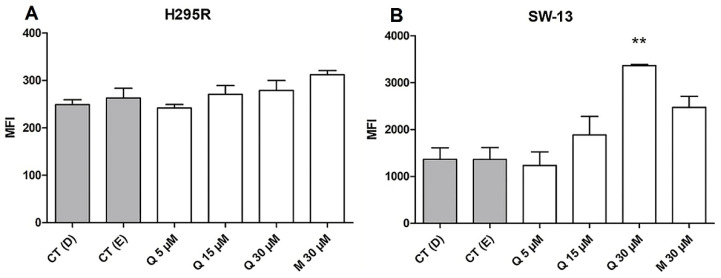
Quercetin and mitotane exerts distinct effects upon intracellular calcium rates. (**A**) Median of fluorescence intensity (MFI) of Fluo-3 AM on H295R; (**B**) MFI of Fluo-3 AM on SW-13 cells. ** (*p* < 0.005) in comparison with respective controls.

The fluorescent probe Fluo-3 AM was used to estimate the intracellular calcium rates, using median fluorescence intensity (MFI) [40]. On H295R cells, both quercetin and mitotane were unable to induce an increase or decrease in Ca^2+^ levels (Figure 7A), whereas the SW-13 cells were positively responsible for quercetin at 30 µM, increasing the MFI (Figure 7B). Calcium ion (Ca^2+^) is associated with apoptosis triggering upon its release from the endoplasmic reticulum (ER) to mitochondria [41]. Since the early and late apoptosis were observed on SW-13 cells, at increased levels when treated with quercetin at 30 µM, the growing pattern of calcium release of those cells at this concentration seems to be correlated with the apoptosis rates.

### 2.6. Quercetin Decreases ROS Generation in the H295R Cells 

**Figure 8:**
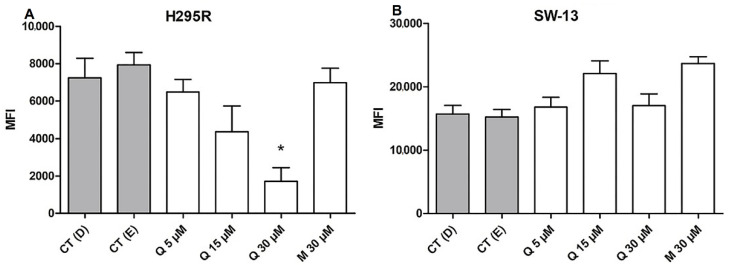
Quercetin performs distinct effects on reactive oxygen species (ROS) generation. (**A**) Median of fluorescence intensity (MFI) of 2′,7′-Dichlorofluorescin diacetate (DCFH-DA) on H295R cells; (**B**) MFI of DCFH-DA on SW-13 cells. * (*p* < 0.05) in comparison with respective controls.

Since the consumption of *Allium cepa* for its antioxidant benefits is remarkable, and quercetin is one of the major flavonoids related to this property [42], it was expected to interfere with the ROS balance. Although the significant difference was appointed only at 30 µM on H295R cells, a reduction pattern on generated ROS was observed with a quercetin dosage increase. ROS rates of SW-13 cells did not express any significant differences (Figure 8A,B).

Targeting ROS balance, up- or downregulating, has been proved as an effective strategy to combat the cancerous cells [43]. Our results may indicate that the antioxidant effect of quercetin on reducing ROS generation on H295R cells may be harmful to this cell, since an increase in the cytotoxicity was also observed, after treatment with the flavonoid at 30 µM. This lowering of ROS rates may also influence the metastatic potential of H295R cells, since it is reported that cancerous cells with higher intracellular ROS rates tend to be more metastatic than others [44], which is the case of the H295R cell line, isolated from a patient with an adrenocortical carcinoma with the aggressive invasive phenotype [45].

## 3. Materials and Methods

### 3.1. Cell Culture and Reagents

The adrenocortical tumor cell lines H295R (ATCC^®^ CRL-2128™), SW-13 (Banco de Células do Rio de Janeiro, RJ, Brazil), and the mammalian epithelial nontumoral cell line MCF-10A (Banco de Células do Rio de Janeiro^®^) were harvested on Dulbecco’s Modified Eagle’s Medium/Ham’s Nutrient Mixture F12 (DMEM F-12) (Sigma-Aldrich, St. Louis, MO, USA), supplemented with 10% (*v*/*v*) of fetal bovine serum (Gibco, Grand Island, NY, USA) and 1% (*v*/*v*) penicillin/streptomycin (Gibco^®^), at a temperature of 37 °C and with 5% of CO_2_. Stock solutions of Quercetin (Q4951; ≥95% HPLC; Sigma-Aldrich^®^), diluted in dimethyl sulfoxide (DMSO), and mitotane (Yick-Vic Chemicals & Pharmaceuticals©, Hong Kong, China), in 95% ethanol, were prepared and diluted to the desired concentration before every experiment. Yellow onion bulbs (*Allium cepa*) from local cultivar were bought in a local market in Curitiba, Brazil, during spring season. Extractions were performed using HPLC-grade ethanol and ethyl acetate (Sigma-Aldrich, St. Louis, MO, USA).

### 3.2. Extraction and Fractionation of A. cepa Components

The bulbs were divided into two groups, inner (In) and outer (Out) layers, which were boiled with ethanol for 2 h at 70 °C. After reducing the extracts to a smaller volume, a fraction of crude extract was firstly collected (In_Cr and Out_Cr), and then 100 mL of the extracts were mixed with 100 mL of ultrapure water and partitioned with 200 mL of ethyl acetate, giving rise to the ethyl acetate (In_EA and Out_EA) and hydroalcoholic (In_HA and Out_HA) fractions. The extracts were dried and stored at −20 °C.

### 3.3. Chromatographic Analysis

The analysis of the *A. cepa* extracts was carried out by high-performance liquid chromatography (HPLC), using a Prominence LC-20A HPLC system coupled with a SPD-20A UV-VIS detector (Shimadzu, Kyoto, Japan). The column used was an Ascentis Express C18-PCP 15 cm × 4.6 mm, particle size 2.7 µm (Supelco Co. Bellefonte, PA, USA). Ultrapure water with 0.05% formic acid (*v*/*v*) as solvent A and acetonitrile with 0,02% formic acid (*v*/*v*), solvent B, was the mobile phase, on the following gradient condition: 1 min 5% B; 5–60% B in 20 min; 60–100% B in 25 min; returning to initial condition (5% B) in 28 min, held for 5 min for system re-equilibration. Separations were developed with the column temperature at 40 °C and a flow rate of 0.5 mL/min. Samples were prepared at 2 mg/mL in methanol–water (1:1, *v*/*v*) and injected at a volume of 2 µL.

### 3.4. Cytotoxicity Assays

The cytotoxicity of *A. cepa* extracts, quercetin, and mitotane was measured initially by the MTT assay (3-(4,5-dimethylthiazol-2-yl)-2,5-diphenyltetrazolium bromide). Briefly, 5 × 10^3^ cells/well were transferred to 96-well plates and left to grow for 24 h, before the addition of both treatments. Two vehicle controls were performed, 0.1% (*v*/*v*) of DMSO and 1% (*v*/*v*) of ethanol 95%, as they were used in the final concentration to dissolve quercetin and mitotane, respectively. Drugs were assayed at a logarithmic scale concentration, at 1 µg/mL, 3 µg/mL, 10 µg/mL, 30 µg/mL, 100 µg/mL, 300 µg/mL, and 1000 µg/mL for *A. cepa* extracts, and 1 µM, 3 µM, 10 µM, 30 µM, 100 µM, and 300 µM for quercetin and mitotane. The drugs were added to the cell lines, with a subsequent incubation for 48 h. The MTT dye (Ludwig Biotecnologia, Alvorada, Brazil) at 0.5 mg/mL was added to each well and then incubated for 3 h. Then, 70 µL of DMSO was added to each well and gently shaken for 20 min, and the absorbance (595 nm) was measured using the microplate reader BioTek El×800 (BioTek Instruments, Winooski, VT, USA).

The second method was the crystal violet assay [46]. Following the same steps of the MTT assay, after 48 h of drug addition, the cells were fixed using paraformaldehyde (4% *g*/*v*), and then 0.25 mg/mL of the crystal violet (Labsynth, Diadema, SP, Brazil) was added to each well (diluted in water) and incubated for 20 min. After two wash cycles with deionized water, 70 µL of the reveal solution (33% of glacial acetic acid in deionized water, *v*/*v*) was added, the plates were gently shaken for 30 min, and then the absorbance (595 nm) was measured using the microplate reader BioTek El×800 (BioTek Instruments). To estimate the cell numbers on each well, a growth curve was performed in parallel, containing a calculated number of cells, to provide a line equation.

### 3.5. Cell Cycle and Proliferation

The cell cycle dynamics were assessed with a 7-Aminoactinomycin D (7-AAD) dye assay. After 48 h of treatment, the cells (5 × 10^5^) were fixed with 70% ethanol at −20 °C for 4 h. Then, the samples were washed with PBS containing 2% (*v*/*v*) bovine serum albumin and incubated with a solution containing 10 µL of 7-AAD (BD Via-Probe™), 20 µL of RNAse A (400 µg/mL; R6148—Sigma-Aldrich) with 0.1% (*v*/*v*) Triton X-100 on PBS [1×] for 30 min, at room temperature, protected from the light. The dye retained on the cell’s DNA was measured by flow cytometry, in a FACSCanto II (Becton-Dickinson, Franklin Lakes, NJ, USA).

The proliferation rates were analyzed using the immunostaining of intracellular protein Ki67. For this, 1 × 10^4^ cells were harvested in 48 well plates for 48 h after the addition of the treatments, then fixed, permeabilized, and blocked with 4% paraformaldehyde, 0.5% Triton X-100 in PBS [1×], and 1% bovine serum albumin in PBS [1×], respectively. The staining was performed using Rabbit Ki67 polyclonal antibody (PA5-16446; Thermo Fisher Scientific, Waltham, MA, USA) and Goat anti-Rabbit IgG Alexa Fluor 488 (A-11008; Life Technologies, CA, USA), both diluted at 1:500, plus nuclear counterstain with 2 µg/mL of Hoechst 33258 (Life Technologies^®^). The imaging and counting of Ki67 positive/negative cells and the total cells were performed using InCell Analyzer 2200 (GE Healthcare, Chicago IL, USA) with 10 randomly distributed fields per well registered by replicate.

### 3.6. Apoptosis and Necrosis Rate

The assay was carried out following the manufacturer’s protocol. After 48 h of treatment, in 5 × 10^5^ cells solution, 1 µL Annexin V FITC (BD Pharmingen, San Diego, CA, USA), diluted in the proper buffer [1×], and 10 µL 7-AAD (BD Via-Probe) were both added and incubated for 15 min at room temperature. The analysis was performed in FACSCanto II (Becton-Dickinson—BD) flow cytometer.

### 3.7. Intracellular Calcium Rate

For this assay, Fluo-3 AM (Invitrogen, Waltham, MA, USA) was used, with adaptations from a previous study [47]. After 48 h of treatment, all cell samples (5 × 10^5^ cells) were trypsinized and incubated with 3 µM of Fluo-3 AM probe, at room temperature and protected from the light, for 30 min. After this first incubation, the samples were centrifuged (300× *g*, 5 min), eluted in PBS [1×], and allowed to stabilize at room temperature for 30 min else. Finally, the samples were analyzed on FACSCanto II (Becton-Dickinson—BD) flow cytometer.

### 3.8. Reactive Oxygen Species (ROS) Measurement

This assay was performed, with some adaptation, as described previously [48]. The cells were treated for 48 h, and then 35 µM of 2′,7′-Dichlorofluorescin diacetate (DCFH-DA) (Sigma-Aldrich^®^) was added to each sample containing 5 × 10^5^ cells, then incubated for 10 min at 37 °C, protected from the light; then, the measurement of fluorescence was performed by flow cytometry on FACSCanto II (Becton-Dickinson—BD^®^).

### 3.9. Statistical Analysis

Statistics were calculated using an unpaired Student *t*-test, with values of *p* < 0.05 considered statistically significant. The data were expressed as mean ± SEM. Tables were made using Microsoft Excel 2019 (Microsoft, Redmond, WA, USA). All statistical analysis was made using GraphPad Prism v. 5.0 (GraphPad Software, San Diego, CA, USA). All flow cytometry analysis was performed using FlowJo^TM^ v. X (FlowJo LLC, Ashland, OR, USA) software, with the fluorometric compensation of dual-color assays calculated on BD FACSDiva (BD Biosciences^™^, San Diego, CA, USA) software.

## 4. Conclusions

To our knowledge, this is the first work comparing the effectiveness of *Allium cepa* extracts and fractions on adrenocortical cell lines, as well the potential of quercetin over mitotane for treating these cancer cell subtypes. The cytotoxicity of *A. cepa* quercetin-enriched fractions showed an interesting potential of the flavonoid on adrenocortical carcinoma treatment. The versatility of quercetin antitumor effects could be observed since the effects on cancerous cell lines were detected in different ways. Being a natural compound with promising usage in oncology and easily obtained from a commonly consumed food, such as *A. cepa*, the potential presented by quercetin as a new option to treat this rare cancer subtype may encourage further investigations. In this regard, a non-clinical investigation is an interesting option to evaluate the potential of quercetin to reduce the tumor mass in an animal-bearing ACC model, which should be useful to study the quercetin bioavailability and metabolism, thus guiding a future use by ACC patients.

## Figures and Tables

**Table 1:** Extraction yield of Allium cepa extracts and quercetin quantification.

Extracts	*Allium cepa* Wet-Weight (g)	*Allium cepa* Dry-Weight (g)	Total Fraction Weight (g)	Extraction Yeld (%) (*)	Quercetin (µg/mg)
**In_Cr**	178.3	34.4	3.9	11.3	0.241 ± 0.002
**In_Et**			3.4	9.9	-
**In_EA**			0.5	1.45	6.16 ± 0.013
**Out_CR**	159.3	36.8	1.3	3.5	7.815 ± 0.136
**Out_Et**			1.1	2.9	1.415 ± 0.011
**Out_EA**			0.2	0.5	108.85 ± 1.130

(*) yield was obtained based on the dry weight.

**Table 2:** Percentage of 7-AAD staining on H295R and SW-13 cells, according to the cell cycle phase.

		G_0_/Sub G_1_	G_1_	S	G_2_/M
**H295R**	**CT (D)**	1.71 ± 0.44	80.23 ± 2.27	4.95 ± 0.82	12.93 ± 1.04
	**CT(E)**	1.92 ± 0.57	79.93 ± 1.27	3.52 ± 0.39	14.63 ± 1.08
	**Q 5 µM**	1.60 ± 0.64	78.47 ± 5.62	4.03 ± 0.73	15.93 ± 4.57
	**Q 15 µM**	2.02 ± 0.57	71.97 ± 4.16	3.95 ± 0.86	21.83 ± 4.12
	**Q 30 µM**	3.44 ± 0.45	65.40 ± 2.19 (**)	5.43 ± 0.79	25.53 ± 2.78 (*)
	**M 30 µM**	3.59 ± 1.17	74.57 ± 2.16	4.21 ± 0.28	17.30 ± 2.29
**SW-13**	**CT (D)**	0.26 ± 0.06	72.93 ± 0.34	4.80 ± 0.22	22.13 ± 0.59
	**CT (E)**	0.28 ± 0.02	74.37 ± 1.57	4.80 ± 0.50	20.37 ± 2.02
	**Q 5 µM**	0.24 ± 0.03	73.77 ± 0.23	5.13 ± 0.51	20.77 ± 0.41
	**Q 15 µM**	1.78 ± 0.26 (**)	69.73 ± 0.26 (**)	3.60 ± 0.75	24.90 ± 0.56
	**Q 30 µM**	7.52 ± 0.65 (***)	64.93 ± 1.41 (**)	4.89 ± 0.90	22.50 ± 1.66
	**M 30 µM**	0.43 ± 0.08	77.33 ± 1.01	4.23 ± 0.64	18.03 ± 1.48

* (*p* < 0.05); ** (*p* < 0.005); *** (*p* < 0.0005), in comparison with respective controls.

**Table 3:** Percentage of Annexin V and 7-AAD staining on H295R and SW-13 cells.

		Necrosis	Late Apoptosis	Early Apoptosis	Live Cells
**H295R**	**CT (D)**	0.27 ± 0.21	1.15 ± 0.85	2.83 ± 0.71	95.77 ± 0.99
	**CT(E)**	0.28 ± 0.23	1.22 ± 0.77	2.56 ± 1.45	95.93 ± 1.39
	**Q 5 µM**	0.26 ± 0.17	0.72 ± 0.38	1.93 ± 1.01	97.07 ± 0.75
	**Q 15 µM**	0.31 ± 0.25	1.89 ± 1.31	4.63 ± 2.59	93.17 ± 2.41
	**Q 30 µM**	0.39 ± 0.36	3.13 ± 2.39	5.71 ± 1.82	90.77 ± 3.01
	**M 30 µM**	0.31 ± 0.27	1.64 ± 1.19	6.58 ± 2.72	91.43 ± 2.62
**SW-13**	**CT (D)**	0.62 ± 0.12	0.64 ± 0.11	2.54 ± 0.66	96.16 ± 0.69
	**CT (E)**	0.61 ± 0.11	0.60 ± 0.17	3.51 ± 0.88	95.26 ± 0.64
	**Q 5 µM**	0.59 ± 0.11	0.67 ± 0.11	3.63 ± 0.76	95.06 ± 0.73
	**Q 15 µM**	1.32 ± 1.02	2.46 ± 0.22 (**)	44.30 ± 5.20 (**)	64.01 ± 11.44 (*)
	**Q 30 µM**	4.63 ± 4.28	15.06 ± 3.75 (*)	71.80 ± 4,80 (***)	26.46 ± 10.46 (**)
	**M 30 µM**	0.89 ± 0.20	1.43 ± 0.04	5.71 ± 1.57	91.93 ± 1.50

* (*p* < 0.05); ** (*p* < 0.005); *** (*p* < 0.0005), in comparison with respective controls.

## Data Availability

Data is contained within the article and supplementary material.

## Supplementary Materials

The following supporting information can be downloaded at: https://www.mdpi.com/article/10.3390/ph15060754/s1, Figure S1: UV spectrum of peaks 1 (**A**), 2 (**B**), 3 (**C**) and quercetin standard (**D**). Figure S2: (**A**) Effect of quercetin (Q) on MCF-10A cells after 48 h of treatment. (**B**) Effect of Mitotane (M) on MCF-10A cells after 48 h of treatment. Figure S3: (**A**,**C**) Measurement of cell count by crystal violet assay on H295R cells after 48 h of treatment with both quercetin (Q) and mitotane (M). (**B**,**D**) Measurement of cell count by crystal violet assay on SW-13 cells, after 48 h of treatment with both quercetin (Q) and mitotane (M). Figure S4: (**A**) Representative images of H295R cells positive stained for Ki67 protein (FITC) on quercetin concentration of 5 and 15 µM; (**B**) Representative images of SW-13 cells positive stained for Ki67 protein (FITC) on quercetin concentration of 5 and 15 µM. Figure S5: (**A**–**F**) Dot plot quad charts for Annexin V and 7-AAD double staining for H295R cells, with respective treatment conditions appointed; (**G**–**L**) Dot plot quad charts for Annexin V and 7-AAD double staining for SW-13 cells, with respective treatment conditions appointed.
