# Supplementary material for: Quercetin-Rich Extracts from Onions (Allium cepa) Play Potent Cytotoxicity on Adrenocortical Carcinoma Cell Lines, and Quercetin Induces Important Anticancer Properties
Source: Pharmaceuticals (Basel). 2022 Jun 16;15(6):754. doi: 10.3390/ph15060754 (PMC9228762; doi:10.3390/ph15060754)

Figure S1: UV spectrum of peaks 1 (A), 2 (B), 3 (C) and quercetin standard (D).

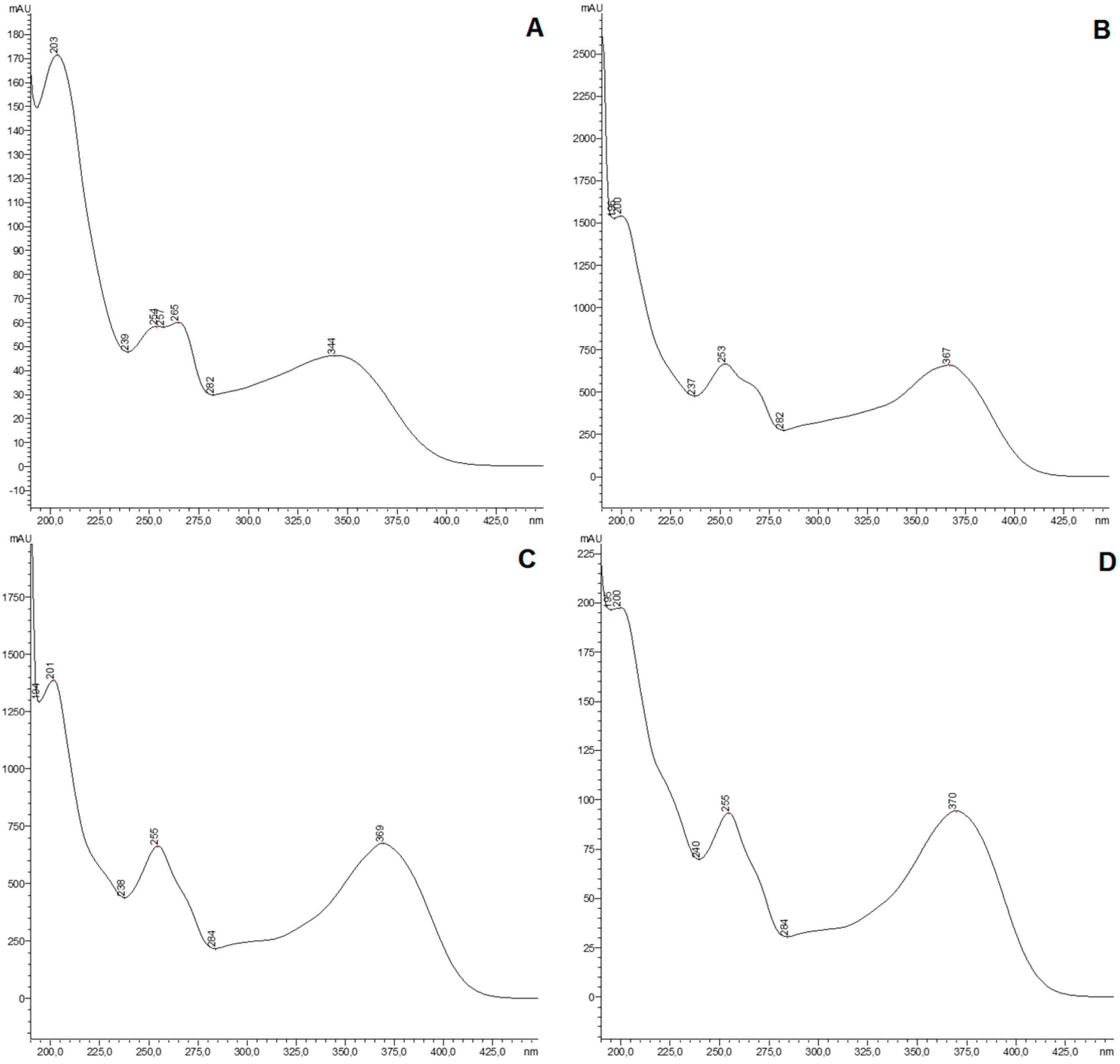

Figure S2: (A) Effect of quercetin (Q) on MCF-10A cells after 48 hours of treatment. (B) Effect of Mitotane (M) on MCF-10A cells after 48 hours of treatment.

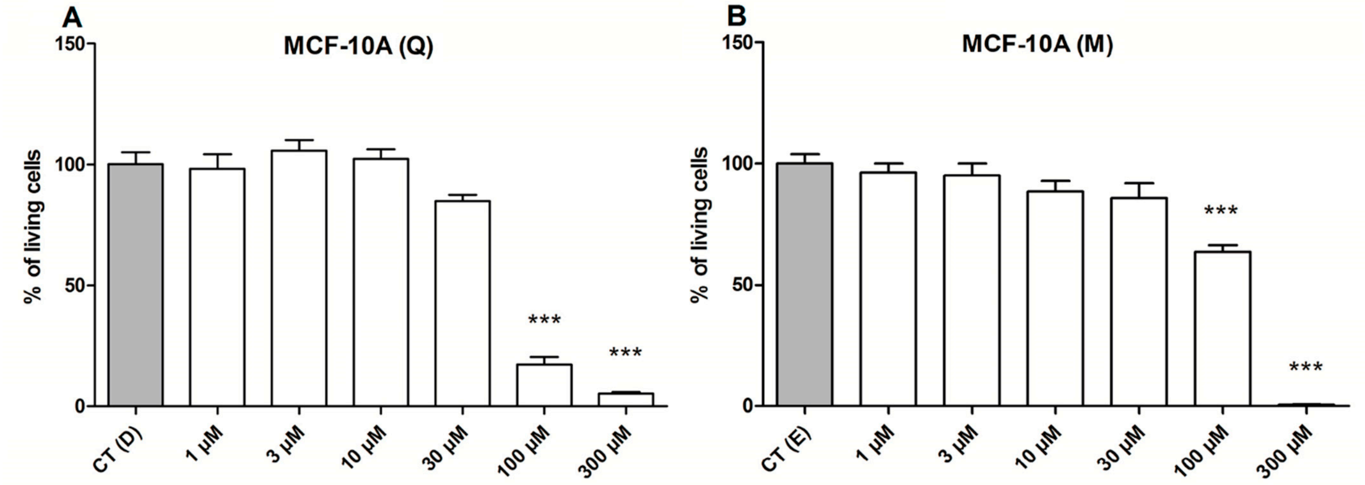

Figure S3: (A and C) Measurement of cell count by crystal violet assay on H295R cells after 48 hours of treatment with both quercetin (Q) and mitotane (M). (B and D) Measurement of cell count by crystal violet assay on SW-13 cells, after 48 hours of treatment with both quercetin (Q) and mitotane (M).

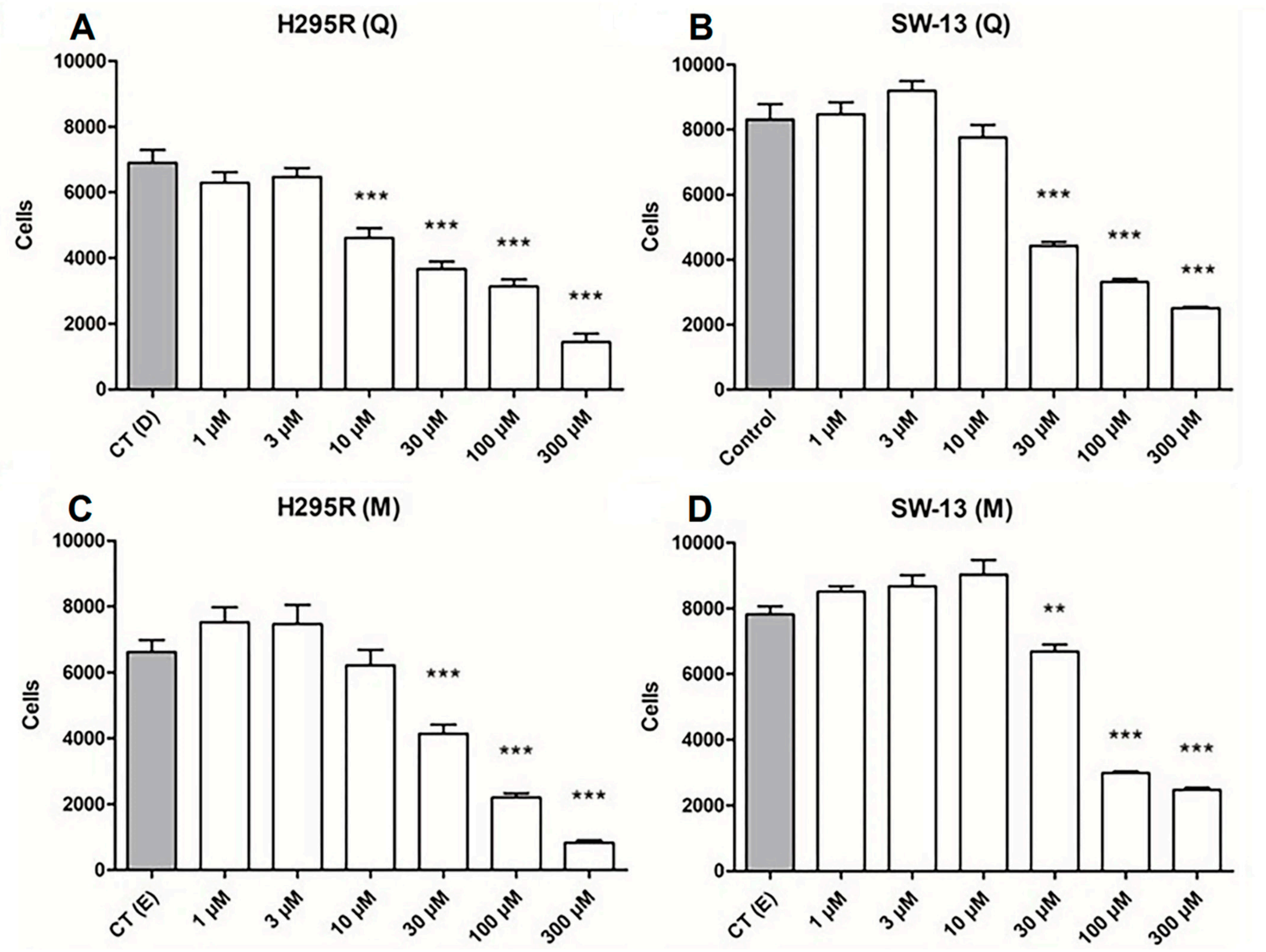

Figure S4: (A) Representative images of H295R cells positive stained for Ki67 protein on quercetin concentration of 5 and 15 µM; (B) Representative images of SW-13 cells positive stained for Ki67 protein on quercetin concentration of 5 and 15 µM.

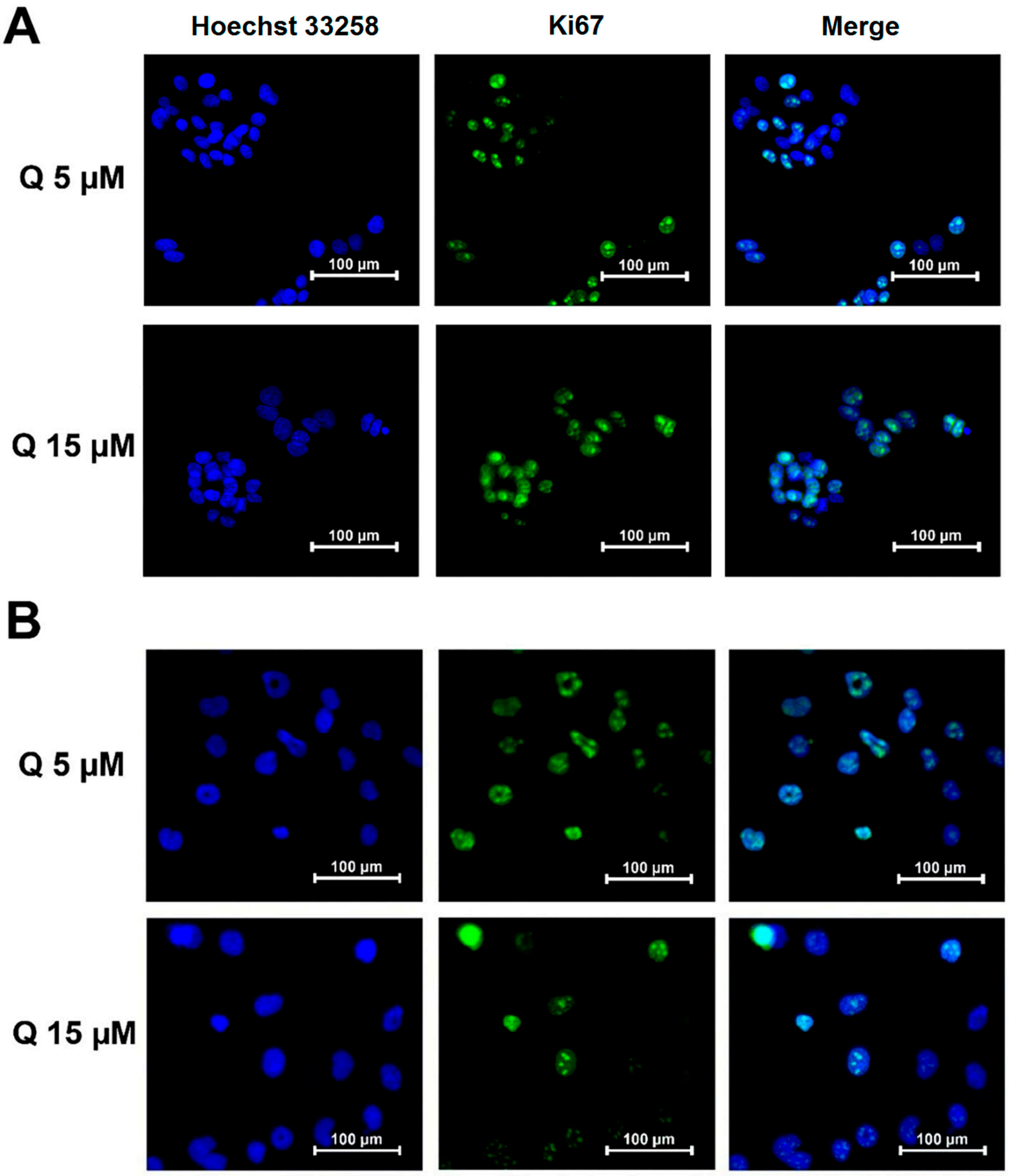

Figure S5. (A, B, C, D, E and F) *Dot plot* quad charts for Annexin V and 7-AAD double staining for H295R cells, with respective treatment conditions appointed; (G, H, I, J, K and L) *Dot plot* quad charts for Annexin V and 7-AAD double staining for SW-13 cells, with respective treatment conditions appointed.

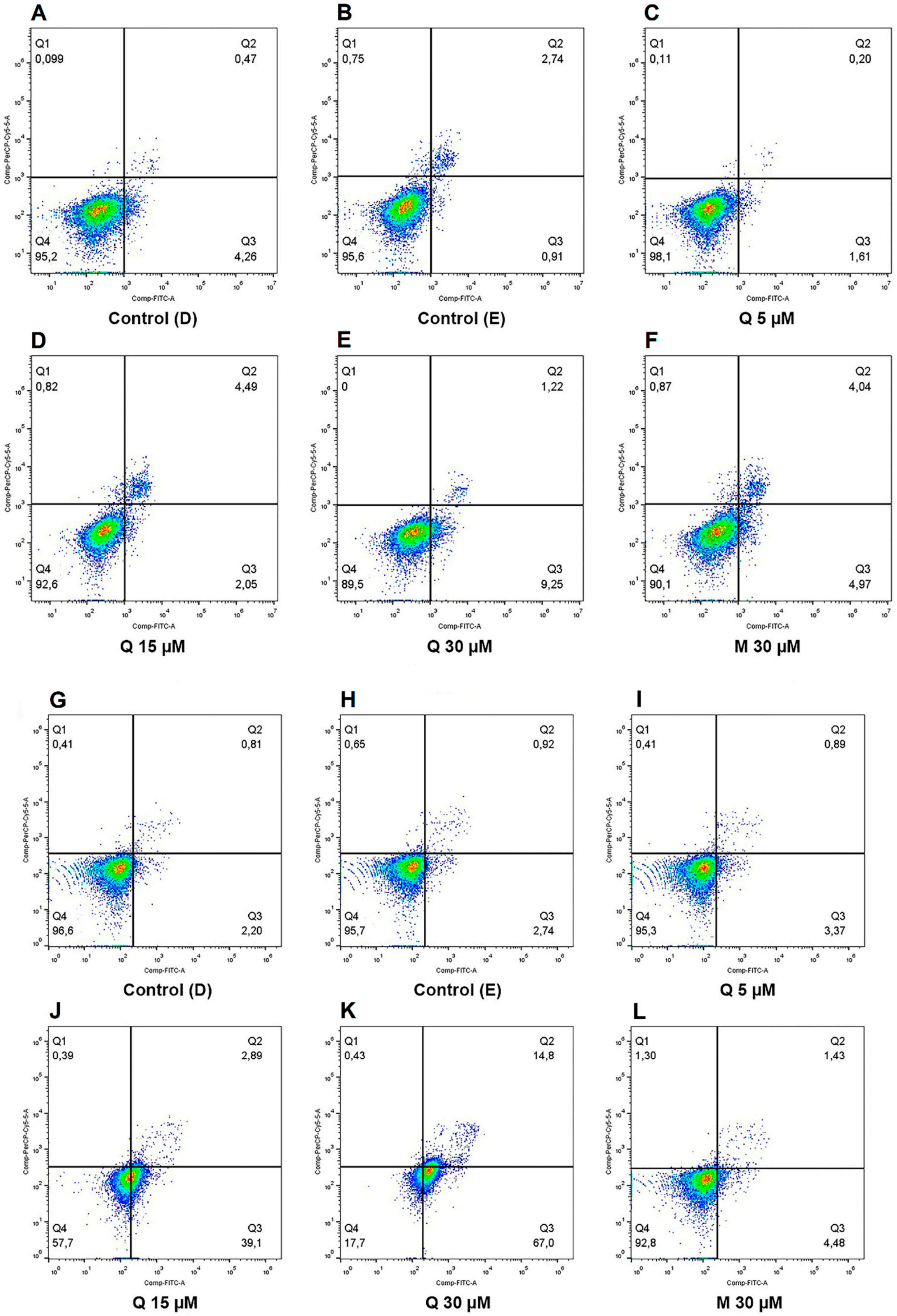

Supplement: Supplementary file 1 [file pharmaceuticals-15-00754-s001.zip › Supplementary Data.pdf]
